# Chatbots to Support People With Dementia and Their Caregivers: Systematic Review of Functions and Quality

**DOI:** 10.2196/25006

**Published:** 2021-06-03

**Authors:** Nicole Ruggiano, Ellen L Brown, Lisa Roberts, C Victoria Framil Suarez, Yan Luo, Zhichao Hao, Vagelis Hristidis

**Affiliations:** 1 School of Social Work University of Alabama Tuscaloosa, AL United States; 2 Graduate Nursing Department Nicole Wertheim College of Nursing and Health Sciences Florida International University Miami, FL United States; 3 Department of Physical Therapy Nicole Wertheim College of Nursing and Health Sciences Florida International University Miami, FL United States; 4 Department of Nursing Nicole Wertheim College of Nursing and Health Sciences Florida International University Miami, FL United States; 5 Department of Computer Science and Engineering University of California, Riverside Riverside, CA United States

**Keywords:** dementia, caregivers, chatbots, conversation agents, mobile apps, mobile phone

## Abstract

**Background:**

Over the past decade, there has been an increase in the use of information technologies to educate and support people with dementia and their family caregivers. At the same time, chatbot technologies have become increasingly popular for use by the public and have been identified as having benefits for health care delivery. However, little is known about how chatbot technologies may benefit people with dementia and their caregivers.

**Objective:**

This study aims to identify the types of current commercially available chatbots that are designed for use by people with dementia and their caregivers and to assess their quality in terms of features and content.

**Methods:**

Chatbots were identified through a systematic search on Google Play Store, Apple App Store, Alexa Skills, and the internet. An evidence-based assessment tool was used to evaluate the features and content of the identified apps. The assessment was conducted through interrater agreement among 4 separate reviewers.

**Results:**

Of the 505 initial chatbots identified, 6 were included in the review. The chatbots assessed varied significantly in terms of content and scope. Although the chatbots were generally found to be easy to use, some limitations were noted regarding their performance and programmed content for dialog.

**Conclusions:**

Although chatbot technologies are well established and commonly used by the public, their development for people with dementia and their caregivers is in its infancy. Given the successful use of chatbots in other health care settings and for other applications, there are opportunities to integrate this technology into dementia care. However, more evidence-based chatbots that have undergone end user evaluation are needed to evaluate their potential to adequately educate and support these populations.

## Introduction

### Background

Over the past decade, chatbot technologies have increasingly been used by people to meet a variety of daily needs, including social and emotional support and information seeking [1]. The term *chatbot* refers to technologies that facilitate human-computer interaction by mimicking natural language conversations with users, either through text or spoken words [2]. Chatbots are also referred to as *conversation agents*, *dialog assistants, or intelligent virtual assistants,* the latter often referring to Google Assistant, iPhone *Siri*, and Amazon *Alexa.* Although interest in chatbots has increased among researchers, software developers, and end users alike, little is known about the efficacy of using chatbots to support people with dementia (which includes Alzheimer disease and related dementias) and or their caregivers. To address this gap in scholarship, the aim of this paper is to systematically review and evaluate the characteristics of chatbots that are currently available on the market and focus on dementia. The findings have implications for advancing technologies that support dementia care and caregiving, which has been identified as a top research priority [3].

### Technologies for People With Dementia and Dementia Caregivers

There has been a growing interest in developing and testing technologies that can improve the quality of life and care for people with dementia and their family caregivers. Indeed, in 2018, the Family Caregiving Institute held a Research Priorities in Caregiving Summit: Advancing Family-Centered Care Across the Trajectory of Serious Illness. Among the 10 research priorities identified, the first 2 are “evaluate technologies that facilitate choice and shared decision making” and “determine where technology is best integrated across the trajectory of caregiving” [3].

Previous research has found that people with dementia have the ability to use a number of technologies designed to fit their needs, including computers [4] and touchscreen technologies [5,6]. Similarly, over the last decade, there has been increasing effort to develop technology-based interventions for dementia caregivers [7]. There is also a growing number of smartphone apps that target the needs of people with dementia and/or dementia caregivers [8,9]. In their systematic review of computer-based cognitive interventions for people with dementia, García-Casal et al [4] concluded that computer-based interventions demonstrated greater efficacy in improving cognition than noncomputer-based interventions. In a systematic review of technology-based interventions for dementia caregivers, it was reported that technology-based interventions often demonstrated efficacy in improving psychosocial outcomes but did not demonstrate efficacy in improving caregiving skills or care self-efficacy. Little is known about the use of chatbots among people with dementia and their caregivers [7].

### Chatbot Technology and Health

Chatbots may use a variety of platforms, including websites, smartphones (eg, Siri), mobile apps (specialized in a domain such as depression or for general purpose, such as WhatsApp and Messenger), SMS text messaging, and in-home smart technologies (eg, Alexa) chatbots. Loranjo et al [10] identified 3 approaches for chatbots to manage dialog: (1) finite state, where there is a predetermined sequence of dialog steps that the user is led through; (2) frame based, where the chatbot asks the user questions and the user responses guide the chatbot through a flow of communication that is not predetermined; and (3) agent based, where artificial intelligence allows the chatbot and user to engage in complex communication. The same review reported that chatbot programs may allow dialog to be led by the chatbot system or the human user; dialog may either be in text or verbal formats.

One of the first established chatbots was programmed in the 1960s, called ELIZA, which was used for text-based psychiatric interviews [11]. This sparked the development of several early chatbots, such as GURU [12], CONVERSE [13], ALICE, and Elizabeth [14]. The renewed interest in chatbots in recent years is largely because of the advancements in deep learning technology [15], which enables agent-based chatbots, and the advancements in voice recognition technology, which have facilitated voice assistants such as Apple’s Siri, Microsoft’s Cortana, Amazon’s Alexa, and Google’s new Assistant [16].

Having been used for customer service, education, website user support, and entertainment, chatbots were found to have promising use in health care [10,17], especially for symptom self-assessment (eg, Babylon Health [18], Sensely, Ada Health, and your.md), and telemedicine [19,20]. Over time, chatbots have been developed and tested to address a number of health conditions and topics, including HIV and sexual health [21], substance abuse, assessment, mental health assessment [22], and weight management [23]. Overall, research on health-related chatbots has demonstrated promising results. In a review of chatbots for psychiatric care, it was reported that chatbots demonstrate efficacy in facilitating psychoeducation and self-adherence to treatment [24].

The benefits of using chatbots for health care have been identified for patients and health care systems alike. For example, chatbots may support patients in treatment management and moral support [25]. For health care systems using telehealth, well-designed chatbots can be a cost-effective way to collect patient data, facilitate patient education, improve engagement with patients, and allocate resources more efficiently [19]. A recent systematic review by Laranjo et al [10] concluded that despite the potential benefits of conversational agents in the health care field, limited research has evaluated the efficacy and safety of this technology.

### Chatbots for Aging and Health

As the state-of-the-art advances, more older adults are using information technologies to meet a number of chronic health needs, such as increasing health self-efficacy, supporting self-care management, engaging in health promotion, and interacting with health care providers [26]. Previous studies have highlighted that auditory chatbots may be especially useful for older adults for health-related communication and information seeking because they operate through voice-driven conversation, which may be helpful for those with low computer literacy [27,28]. Chatbots, such as virtual assistants, have been used by older adults for many purposes. A previous study showed that voice-activated personal assistants were used as medication reminders for older adults [28]. Another study also indicated the application of chatbots to follow up older patients with cancer receiving chemotherapy at home [25].

In the case of dementia care and caregiving, information technologies have specifically been identified as a potential way to overcome the existing challenges of accessing education and emotional support [29,30]. Caregivers of people with dementia often experience depression, burden, and poor health outcomes because of lack of emotional and caregiving support, knowledge, and coping strategies for the complexity of dementia care [31]. This can have a negative impact on the quality of care and outcomes for people with dementia [31]. Information technology can overcome existing barriers to education and support for caregivers who are unable to attend in-person services or need flexible scheduling of services [8]. Preliminary studies on information and other mobile technologies have also demonstrated positive benefits for caregivers [8,32]. Mobile information technologies targeting people with dementia have mostly focused on improving memory and engagement [5]. Given the previous evidence presented on the benefits of using chatbots among older adults and in health care in general, they may pose unique benefits in providing education and support to people with dementia and their caregivers. They may especially be beneficial for those living in rural communities, who may face the challenge of distance and limited resources for interacting with providers and services [33]. It should be noted that there are commercially available apps that use chatbots for making clinical assessments of people with dementia [34]. However, many of these are designed to be used with the assistance of a professional and are not focused on education and support for care at home.

### Need for Critical Review

Many of the identified uses for chatbots, such as self-assessments and health education, are potentially helpful in educating and supporting people with dementia and their caregivers. Advancing such technologies for the purpose of dementia care is critical, given that states are projected to increase the incidence of neurodegenerative diseases by up to 33% between 2019 and 2025 [31]. However, little is known about how chatbots may be useful for dementia care and caregiving. To address this gap, this study systematically identified and evaluated existing chatbot apps that are available on the market to educate and support people with dementia and their caregivers.

## Methods

### Overview

Previous studies on smartphone apps informed the methodology for evaluating existing chatbots [35]. A review of chatbot apps that focus on dementia was conducted between December 2019 and April 2020. Given the lack of chatbot app research in the academic literature, 3 investigators (NR, YL, and ZH) independently conducted searches on Google Play Store, Apple App Store, Amazon’s Alexa Skills, and the internet. Key search terms used alone or in combination included “Chatbot,” “Virtual Assistant,” “Alzheimer’s,”” dementia,” “health,” and “caregiver.”

Chatbot apps were included for evaluation if they were programmed to engage in interactive dialog with the technology, verbally or through text; were focused on the topic of dementia; were designed for use by dementia caregivers and/or people with dementia; were available for download and use; were available in English; and were not games. Chatbots were excluded if they were programmed so that users chat with a live person, did not have content related to dementia, were designed to be used by providers, were not currently available for use, were not available in English, or were games. A total of 6 investigators were involved in evaluating apps for inclusion or exclusion criteria, and at least two investigators reviewed each app independently.

### Critical Review

**Figure 1:**
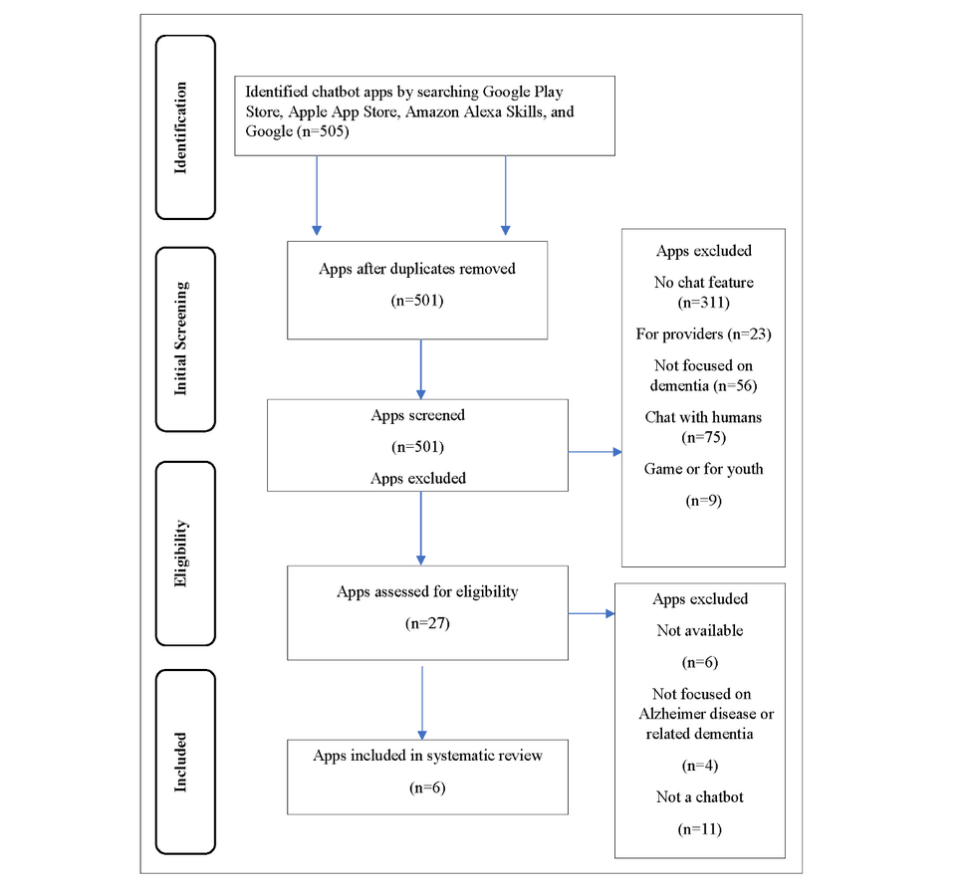
Flowchart of the chatbot apps searched and the screening procedures conducted using the PRISMA (Preferred Reporting Items for Systematic Reviews and Meta-Analyses) guidelines.

The evaluation tool of identified chatbots was developed using the framework developed by Radziwill and Benton [36] for evaluating chatbots, which is grounded in the computer science literature. The characteristics assessed are shown in Figure 1 and include *efficiency*, which included the category of *productivity*; *effectiveness*, which included the categories of *functionality* and *humanity*; and *satisfaction*, which included the categories of *affect* and *ethics and behavior.* A total of 3 authors (ELB, LR, and CVFS) evaluated each item of the assessment and graded the item using the following scale: ✓ (check), indicating that the evaluators could observe the attribute and/or the app-performed attribute function; — (em dash), indicating that the attribute or function was nonfunctional or inconsistent in functionality when accessed, and N/A (not applicable), indicating that the attribute did not apply to the stated functions of the app. The authors then compared their grades for each item and discussed discrepancies until an agreement was reached. A thematic analysis then was conducted based on the trend in scoring on the assessment across chatbots. All observations made by the team about the chatbot were based on their performance when evaluated in April 2020.

## Results

### Overview

The results of the search strategy are shown in Figure 1. Overall, 6 chatbot apps targeted the topic of dementia: 1 text-based mobile app and 5 Alexa Skills voice apps. It should be noted that the text-based mobile app (CogniCare, CogniHealth Ltd) was associated with one of the Alexa Skills apps, and these apps were the only ones identified to have an associated website. Half of the apps focused on education about dementia, whereas the other half focused on memory and reminiscence. The educational content provided through the apps was generally about the epidemiology and symptoms of dementia and less focused on caregiving skills and activities. None of the apps made claims about the expected outcomes of using the app, and no peer-reviewed research was identified for any of the apps. General information about the apps reviewed can be found in Table 1, and the assessment of the apps can be found in Table 2. The assessment of the apps took place in May 2020; therefore, the characteristics of the app presented here may have changed since then.

**Table 1:** General information about the chatbot apps.

App name	App ID	Developer name	Operating system	Downloads (N)	Price	Privacy statement
CogniCare mobile app	1	CogniHealth Ltd (in partnership with Alzheimer Scotland)	Android and iOS	≥1000	Free	Yes
CogniCare (Alexa Skills version)	2	CogniHealth Ltd (in partnership with Alzheimer Scotland)	Alexa Skills	Not reported	Free	Yes
My Life Story	3	Z55-Studios	Alexa Skills	Not reported	Free	Yes
Dementia Types	4	T Tillman	Alexa Skills	Not reported	Free	Yes
Build Your Brain Power	5	Fire Up Your Goals LLC	Alexa Skills	Not reported	Free	Yes
Everything Memory	6	jaimiles23	Alexa Skills	Not reported	Free	Yes

**Table 2:** Assessment tool informed by the framework developed by Radziwill and Benton and evaluation of apps by the research team.

Category, Quality attribute^a^					App IDs							
					1		2		3	4	5	6
**Efficiency**												
	**Performance**											
		Robustness to unexpected input		✓^b^		—^c^			✓	—	—	—
		Avoids inappropriate utterances and is able to perform damage control		N/A^d^		—			✓	—	—	N/A
		Effective function allocation and provides appropriate escalation channels to humans		—		N/A			✓	—	—	N/A
**Effectiveness**												
	**Functionality**											
		Accurate speech synthesis		N/A		—			✓	—	✓	✓
		Interprets commands accurately		✓		—			✓	—	✓	✓
		General ease of use		✓		—			✓	✓	✓	✓
		Executes requested tasks		✓		—			—	✓	✓	✓
		Contains breadth of knowledge and is flexible in interpreting it		✓		—			N/A	✓	✓	✓
	**Humanity**											
		Passes the Turing test		N/A		—			✓	—	—	—
		Convincing, satisfying, and natural interaction		N/A		—			✓	—	—	✓
		Able to respond to specific questions		✓		—			N/A	—	N/A	N/A
		Able to maintain themed discussion		N/A		—			N/A	✓	N/A	N/A
**Satisfaction**												
	**Affect**											
		Provides greetings and conveys personality		N/A		✓			✓	✓	✓	✓
		Gives conversational cues		N/A		—			N/A	✓	✓	✓
		Exudes warmth and authenticity		N/A		✓			✓	✓	—	✓
		Makes tasks more fun and interesting		✓		—			✓	✓	—	✓
		Entertains and/or enables participant to enjoy the interaction		✓		—			✓	✓	—	✓
	**Ethics and behavior**											
		Ethics and cultural knowledge of users		✓		✓			—	—	—	—
		Protects and respects privacy		✓		✓			✓	✓	—	✓
		Sensitivity to safety and social concerns		✓		✓			—	—	—	✓
		Trustworthiness (of information source)		✓		✓			—	—	—	—
		Awareness of trends and social context (in language and conversation)		—		—			—	—	—	—

^a^Apps were evaluated in May 2020.

^b^Evaluators could observe the attribute and/or the app-performed attribute function, as described earlier.

^c^The attribute or function was nonfunctional or inconsistent in functionality when accessed.

^d^N/A: not applicable.

### Efficiency in Performance

For all apps, their functions and features were described in detail on Google Play Store or Alexa Skills pages. A total of 2 apps (app number 4 and 6) had more detailed instructions for getting started with using the apps than the others. One app (app number 2) offered an extensive guide to using the app with Alexa, although this guide was found on the developer’s website and was not linked or listed on Amazon.com, where the app could be found and accessed. On Amazon.com, there was only a list of voice commands in speech bubbles that suggested questions to ask the chatbot. This posed challenges for the evaluators who initially accessed the apps for evaluation. The mobile text–based chatbot (app number 1) was the easiest to get started.

The evaluators also faced linguistic challenges when initially accessing the functions of the voice chatbot apps. For instance, some instances of Alexa’s voice recognition were not flexible with the user’s pronunciation of commands or required a specific command to get started in the chatbots, which was not immediately understood by the researchers. This may also be the reason that for voice chatbots, the evaluators noted that they would perform some of their described features, but not others. Only 1 chatbot (number 3) performed well on all 3 performance attributes. This was an audio chatbot that went through a series of questions about the user’s life history, where the information was programmed into the app and the chatbot would tell the user their life story on command. For 3 of the chatbots, not all performance attributes were applicable.

### Effectiveness

#### Functionality

In general, the chatbots were evaluated more favorably for their functionality, compared with the other categories assessed. In total, 5 of the 6 chatbots were found to be easy to use once the researchers were able to get the programs started. Of the 5 chatbots designed to educate about dementia, 3 were found to have a breadth of knowledge and flexibility in interpreting information.

#### Humanity

All apps demonstrated the ability to interact with users through voice or text responses to users’ input. Although users found the apps’ responses to their input to be understandable and logical, their responses were perceived to be more automated than natural conversation. Only 1 app (app number 3) passed the Turing test, which is the extent to which one can tell the difference between conversing with a human and the chatbot. The researchers also found that the programmed dialog in all the chatbots was limited in scope, which made extended language exchange between the user and chatbot to also be constrained. For example, 1 chatbot (app number 4) provided a definition for each type of dementia, but there was no additional content for a true back-and-forth exchange.

### Satisfaction

#### Affect

In general, the evaluators assessed the chatbots as having features aimed at increasing the user’s enjoyment while using them. For instance, 1 app (number 3) played calm music in the background while the users accessed its other features. All audio chatbots provided greetings and/or conveyed personality. Only 3 chatbots appeared to provide conversational cues to facilitate the user’s ongoing engagement. For example, 1 chatbot would instruct the user to “Say, ‘tell me more’” after it completed its dialog (app number 4). A total of 3 apps were evaluated as having the potential to make caregiving tasks more enjoyable or interesting.

#### Ethics and Behavior

Safety and privacy protection were evaluated for chatbot apps. In total, 5 apps had privacy statements describing the terms of use and security of users’ input. In all the privacy statements, the developer confirmed that none of the users’ information would be shared with third parties, unless the user gave permission to do so. In total, 2 apps (app number 2 and 6) included statements in their app descriptions that the chatbot was not a substitute for medical care or advice. In terms of trustworthiness of information, 4 apps were created by private developers. Two of the chatbot apps (app number 1 and 2) were developed by the same entity, which partnered with the nongovernmental organization Alzheimer Scotland to develop both the apps. It should be noted that since the time of review of the chatbots, the organization has also partnered with the University of Edinburgh for development. Hence, these apps were viewed as having the highest perception of trustworthiness.

Although the apps had content and functions that were deemed interesting and useful, some of the limitations in programmed content (as described earlier) presented challenges in this category as well. For instance, none of the apps were available in languages other than English, although 1 app (number 2) listed that their app was available in English from 5 different countries. The evaluators were not able to assess whether this resulted in language differences (eg, idioms and pronunciations) that were country specific. None of the apps were programmed to respond to patterns or the social context of user input.

## Discussion

### Principal Findings

At the time of this review, it was found that commercially available chatbot apps for people with dementia and their caregivers appeared to be in an early stage, with only 6 identified apps that met the search criteria across Google Play Store, the Apple Store, Amazon Alexa Skills, and the internet. All but one of these chatbot apps was the Alexa Skills app. Although chatbots, and specifically intelligent voice assistants, have been suggested as a potential source of assistance for older adults in general [37], their assessment indicates that people with dementia and their caregivers may experience challenges in using them. For instance, most apps had limitations in their programmed content, which created challenges when evaluators did not use specific commands. This may create barriers for populations with cognitive impairment or populations that use English as a second language. It may also create challenges for those who are not already familiar with chatbot technologies. Hence, people with dementia and caregivers could become frustrated when chatbots do not function as expected.

Limited program content also created challenges in having extended and/or varied conversations between users and chatbots. Owing to the complexity of dementia and variations in symptoms, this could limit the amount of education and support provided to a diverse population of people with dementia and their caregivers. This could impede the use of chatbots for information seeking and support. However, the hardware required for intelligent voice assistant platforms, such as Alexa, is recognized as having potential benefits for people with dementia and their caregivers. For instance, Alexa Skills relies on voice for navigation, which may be easier for older adults than typing, and does not require technical accessories, such as a mouse or keyboard. Hence, it may be worthwhile to further investigate how chatbots can be designed with user-friendly features for older adults, especially those who may have cognitive or memory impairments.

Several of the chatbots reviewed included educational information, although references were not provided for the informational sources; therefore, it was unclear whether health-related information provided by the chatbots is evidence based or whether information has been updated as needed. Designers should consider providing and using peer-reviewed references used in programming chatbots. In addition, although chatbot apps appeared to focus on education about dementia and engagement, the apps did not offer much information on caregiving skills and activities. However, the guidebook found for the CogniCare Alexa Skills chatbot reported that such content was being planned for the future. Such content would increase the usefulness of the chatbots for caregivers, who often feel isolated and have low caregiving self-efficacy.

### Humanity Versus Usefulness

We found that most chatbots did not pass the Turing test, which mainly means that they were unable to keep the conversation going while pretending to be human. We note that acting human may not be enough if the chatbot does not achieve its goals of educating or helping caregivers or patients. Most early research on chatbots has focused on the humanity aspect [11], whereas more recent research has focused on how to create goal-oriented chatbots, which understand the user and complete a transaction, such as making an appointment or answering a question. Much of this recent work assumes that there is a data set of conversation on the domain to train, which is not the case for dementia care, where there are no available conversations between patients and providers. Nevertheless, as more artificial intelligence tools are created, we expect that the responsiveness of dementia chatbots will improve as well.

### Need for Evidence-Based Chatbots for Dementia Care and Caregiving

It is important to explore how chatbots may be used to educate and support people with dementia and their families. There are many potential chatbot functions that those affected by dementia may value depending on their preferences and needs. For instance, given the previous applications of chatbots to adult learning, chatbots could be used to educate and train caregivers on a variety of topics, especially those who feel isolated [38]. Chatbots can also be programmed so that caregivers can take self-assessments and receive evidence-based feedback or be linked with resources. For people with dementia, designers may consider how evidence-based cognitive training and other evidence-based programs can be administered via a chatbot [39].

When considering people with dementia as targeted end users, designers should also consider that there is a continuum of severity in dementia symptomology and assess how content and functions may be appropriate for different stages of disease progression or adaptable to be used as symptoms progress. For example, people with mild dementia may look for and benefit from features that support independence or memory-building activities. However, people in later stages of dementia and their caregivers may prefer features that promote engagement and reminiscence, such as a recorded message from a loved one or a favorite song. Listening to music can be a shared enjoyable activity by the care recipient and caregiver, and music therapy has been found to be therapeutic in managing behavioral disturbance in people with dementia [40].

Although there are opportunities to advance the state of the science on dementia-focused chatbots in general, it is important that such chatbots should be evidence based. It is unclear whether the chatbots included in this review underwent empirical user testing. It should be noted that since conducting the chatbot review, the developers of CogniCare have published a peer-reviewed article on the development of their apps [41], although this article did not report outcome findings for users. Overall, this underscores the importance of developing chatbot technologies that undergo extensive user testing, given that the technical literacy required to use chatbots can pose a barrier to older adults’ use and acceptability [25]. This is very important for chatbots designed to be used within the context of health care, given the previously stated concerns that input inflexibility of a particular chatbot could pose risks to patients [10]. This point also relates to the quality of the content programmed in a chatbot. For instance, it is unclear the extent to which educational material provided through the chatbots assessed for this study were based on evidence from the literature on dementia.

### Opportunities for Integrating Chatbots Into Dementia Care Delivery

There are particular potential benefits of integrating chatbot technology into dementia care settings. People with dementia and their caregivers are at an increased risk of social isolation, especially for people with dementia who are in long-term care settings. The potential for chatbots for socially isolated populations has been particularly evident during the COVID-19 pandemic, where the Center for Disease Control and Prevention launched its Clara Bot app as an interactive way to educate the public and provide emotional support [42]. Integrating chatbots into dementia clinical settings could also potentially reduce the burden on staff by offering vetted information to patient and caregiver populations, which could reduce calls and questions for clinical staff. It should be noted that the quality of the information provided by chatbots in this review was not fully evaluated, and it is unclear if they provided vetted information. In addition, the limitations of the chatbots critically reviewed in this study, such as the need for specific phrases and pronunciations, highlight the need for developing health care chatbots that support diverse patient populations. For instance, dementia chatbots that are flexible in their understanding of varying accents and dialects will increase their acceptability and ease of use.

A key obstacle in integrating chatbots into care delivery is the Health Insurance Portability and Accountability Act (HIPAA) compliance needed for the chatbot to be able to exchange information with a health care entity (covered entity). Fortunately, Amazon Alexa has recently begun offering HIPAA-compliant skills [43]. Mobile apps can also be HIPAA compliant, but this is not the case for chatbots deployed on existing platforms, such as Messenger, SMS text messaging, or WhatsApp [44].

### Limitations and Future Needs

There are limitations to this critical review that should be considered when interpreting the results. Despite the extensive search and review process used to identify chatbots, it is possible that there may be additional chatbots that address dementia but were not identified. In addition, there are no standardized measurement tools for chatbot technology. The authors initially attempted to conduct this review using the widely used mobile app rating scale [45]. However, too many items did not apply to chatbots. It should be noted that the evaluation tool used for the review developed by Radziwill and Benton [36] is heavily grounded in evidence on chatbots.

### Conclusions

Although chatbots offer the potential for an engaging technology that can benefit people with dementia and their caregivers, more work is needed to develop evidence-based chatbots that are easy to use for these populations. Future research in this area should involve interdisciplinary scientific teams that have the expertise to develop a high-performing chatbot technology as well as expertise in dementia or health education.

## Abbreviations

HIPAA: Health Insurance Portability and Accountability Act
